# Investigating Factors Associated With not Reporting Medical Errors From the Medical Team’S Point of View in Jahrom, Iran

**DOI:** 10.5539/gjhs.v6n6p96

**Published:** 2014-07-15

**Authors:** Zohreh Badiyepeymaie Jahromi, Nehleh Parandavar, Saeedeh Rahmanian

**Affiliations:** 1 Department of Nursing and Midwifery, Jahrom University of Medical Sciences, Jahrom, Iran

**Keywords:** medical team, medical error, reporting error, patient safety

## Abstract

**Background:**

medical errors as a problematic fact in healthcare systems can increase patient’s safety if reported. This article tried to determine several factors associated with not reporting medical errors from medical team’s points of view.

**Methods:**

300 staff working in different parts of educational hospitals affiliated to Jahrom University of Medical Sciences including nursing, midwifery, paramedical and medical groups participated in this descriptive study using census method (2013). Data collection was performed using a researcher-made questionnaire including 31 items regarding four areas: medical teams, managers, errors and patients.

**Results:**

The mean score of factors related to errors, mangers, medical teams, and patients’ scope was 2.68 ± 0.79, 2.63 ± 0.72, 2.53 ± 0.66, 2.41 ± 0.87, respectively. In medical teams’ points of view, errors and managers were among the important factors for not reporting professional errors. The most important factors in professional errors were related to severity and emergency of errors (2.73 ± 0.97), and managers’ focus on wrongdoers instead of noticing systematic factors of errors (3.00 ± 1.01). In medical teams, fear of legal prosecution by patients or their relatives (2.87 ± .97), and in patients, unawareness of errors (2.67 ± 1.08) was reported as the most effective factors.

**Conclusion:**

Factors related to errors and managers were more important than other reasons. Therefore, educating medical teams on recognizing errors and managers’ proper reactions in case of occurring or reporting errors seem to be necessary.

## 1. Introduction

Error means wrong position and mood in personal action and judgment (Nikpeyma & Gholamnejad, 2009). This is an inseparable part of human’s life. Most errors are originated from psychological processes and behavioral consistencies which can cause correct behavior. As everybody may commit errors, medical teams are not excepted, regardless of their proficiency, commitment, and accuracy in doing their professional duties (Hashemi, 2008). This error is an action term, due to which the programmed chain of physical and conceptual activities in health will fail and it is not regarded to be by chance (Nikpeyma & Gholamnejad, 2009).

Reports showed that medical service is the most hazardous action. The rate of mortality due to medical errors is higher than breast cancer, air crashes, traffic accidents, and HIV (Haidar Pour et al., 2011). In spite of improvement in technology and healthcare skills, lots of patients were hurt or died because of medical teams’ errors which, in some cases, cost a lot (Hashemi, Nikbakht Nasrabadi, & Asghari, 2012). Unlike other jobs, it has irrecoverable results (Nikpeyma & Gholamnejad, 2009). These errors are the main facts in healthcare system. We can use these items for evaluating patients’ safety because of the prevalence of errors in hospitals (Mohammadnejad, Hojjati, Sharifnia, & Ehsani, 2009). Nursing group in caring services is responsible for most of medical teams’ errors; investigating pharmaceutical errors is of great importance because of high rate of mortality and hospital costs (Guchelaar, Colen, Kalmeijer, Hudson, & Teepe-Twiss, 2005).

Errors show problems in the organization such as a lack of patient’s safety culture and unfavorable occupational status of medical teams. We can report and record errors for pursuing and educating ways of preventing them. Therefore, reporting professional errors is necessary for patient’s safety and emergency care centers (Hashemi et al., 2012).

Unfortunately, a lot of anxieties hinder medical teams to report errors (Hashemi, Nikbakht, Nasrabadi, & Asghari, 2011). That is why there are not any clear classified data about the cause and type of errors (Nikpeyma & Gholamnejad, 2009). Reporting and recording medical team’s errors are useful for organizational programs (Haidar Pour et al., 2011). As other articles have focused on errors by nursing groups, this article tried to investigate other points of view in medical groups including doctors, midwives and other paramedical groups.

### 1.1 Research Aim

This descriptive research aimed to show several factors related to not reporting professional errors from the medical team’s point of view in 2013.

## 2. Method

### 2.1 Participants

300 staff working in different parts of educational hospitals affiliated to Jahrom University of Medical Sciences in south west of Fars province (including nursing groups, midwifery and paramedical subcategories) participated in this descriptive research (2013). The inclusion criteria were: one year of medical care service and tendency to take part in the study.

### 2.2 Ethical Considerations

The research ethics committee of Jahrom University of Medical Sciences confirmed the ethical conduct of the study. We explained our goals to the study participants and assured them that the information would be kept confidential and the questionnaires would be completed without any mention of their names. The information of people who were not willing to take part in the research was not gathered.

### 2.3 Data Collection

Data collection was done using a questionnaire consisting of prevalent factors of not reporting and recording professional errors.

### 2.4 Questionnaire

we provided a questionnaire consisting of demographic characteristics (age, gender, years of service and current job position and a special part related to not reporting professional errors in four scopes of medical teams, managers, errors and patients (13 questions related to medical teams, 10 questions related to managers, 3 questions related to errors, and 5 questions related to patients) based on Likert’s 5 point scale from completely agree to completely disagree (0-4). For checking reliability and validity of the questionnaire, we derived our items from valid sources and recent studies. 31 questions related to not reporting professional errors were included in the questionnaire. About 10 instructors of Jahrom University of Medical Sciences observed and confirmed our questionnaire to be correlated. 20 nurses completed the questionnaires, and Cronbach’s coefficient was estimated to be 0.91. Cronbach’s coefficient was evaluated for the medical teams (0.83), managers (0.82), error (0.74), and patients (0.77).

### 2.5 Procedure

All the questionnaires were distributed and gathered collectively in morning, afternoon, and night shifts by the researcher herself. Therefore, the study participants could freely answer them without any restrictions. Each questionnaire needed about 10-15 minutes to be answered.

### 2.6 Data Analysis

Descriptive statistics were reported as frequencies, means, and standard deviations. Correlation analysis was used to evaluate the relationship between several factors of not reporting errors and demographic data.

## 3. Results

400 questionnaires were distributed, and 300 questionnaires were finally gathered. The mean response rate was %75. The mean age and years of service was 28.80 ± 6.48 and 6.92 ± 6.35, respectively. 210 (70%) participants were female and 186 (57.6%) were in nursing and midwifery posts (Table 1).

**Table 1:** Characteristics of the participants (N=300)

Demographic characteristics		N (%)
gender	Male	90(30)
	Female	210(70)
Job Position	medical	26(7.8)
	Nursing and midwifery	186(57/6)
	Paramedical	78(24)

The mean score related to reasons of not reporting professional errors, included medical teams (2.53 ± 0.66), managers (2.63 ± 0.72), errors (2.68 ± 0.79) and patients (2.41 ± 0.87). Reasons related to managers and errors were the most important ones for not reporting professional errors.

Among the factors related to medical teams, fear of legal prosecution 2.87 ± 0.97 (69.1%), concerns over inadequacy 2.75 ± 1.01 (63.9%), fear of losing one’s position 2.73 ± 1.01(66.9%) and worrying about grandiose errors from their colleagues 2.75 ± 1.09 (61%) were the most important reasons for not reporting and recording professional errors.

Among the factors related to managers, their focus on wrongdoers rather than the results of occurring errors 3.00 ± 1.01 (74.6%), managers’ discrimination toward wrongdoer 2.76 ± 1.09 (62.3%), and their improper reaction 2.70 ± 1.07 (62%) had the highest scores among the reasons for not reporting errors.

Among the factors related to errors, severity and importance of errors 2.73 ± 0.97 (63%), types of errors such as errors of procedures, bad performance, delayed cares 2.65 ± 0.98 (60.2%), and medical team did not know a clear definition of errors 2.63 ± 0.98 (58.8%) had the highest scores among the reasons for not reporting errors.

Among the factors related to patient’s, their uncertainty about errors 2.67 ± 1.08 (62.2%), patient’s critical situation 2.43 ± 1.11 (55%), prognostication of death 2.38 ± 1.15 (50.5%) were the most important reasons for not reporting and recording professional errors (Table 2).

**Table 2:** Relative and cumulative frequency, mean score and standard deviation of not reporting errors

M ± SD	Disagree and Completely disagreement N(%)	No opinion N(%)	Agreement and Completely agreement N(%)	Scope and phrase
**Medical team scope**				**2.53 ± 0.66**
Fear of patients legal pursue	221(69.1)	68(21.2)	31(9.6)	2.87 ± 0.97
Fear of position	214(66.9)	61(19.1)	45(14.1)	2.73 ± 1.01
Fear of in adequacy	205(63.9)	75(23.4)	41(12.8)	2.75 ± 1.01
Fear of patient’s blame and his family	175(54.8)	82(25.7)	62(19.4)	2.54 ± 1.08
Fear of colleague’s blame	171(51.3)	95(29.6)	55(17.1)	2.56 ± 1.10
Fear of amplify errors by colleagues	193(61)	79(24.6)	49(15.3)	2.70 ± 1.09
Fear of errors influence in their income and premium	155(48.6)	66(20.7)	98(30.7)	2.26 ± 1.20
Fear of negative attitudes against medical team’s member	150(45.0)	109(34.1)	61(19.0)	2.42 ± 1.06
Fear of distrust against one group of treatment	57(50.8)	87(27.1)	71(21.1)	2.41 ± 1.08
Lack of responsibility and professional responding in medical team’s member	153(47.8)	80(25)	87(27.2)	2.31 ± 1.17
Not observed Long-term effects of error by the medical team	175(54.6)	80(25)	65(20.3)	2.49 ± 1.07
Low information	171(53.6)	89(27.9)	59(18.5)	2.46 ± 1.08
Low proficiency of reporting	160(50.3)	98(30.8	67(18.8)	2.39 ± 1.09
**Managers scope**				**2.63 ± 0.72**
Managers worry about defame of medical clinic	181(56.9)	65(20.4)	72(22.7)	2.51 ± 1.13
Uniformity of errors in clinic	143(45.1)	72(22.7)	102(22.2)	2.24 ± 1.25
Protrusion of pressure and responsibility between members	168(52.9)	83(26.1)	67(21.1)	2.50 ± 1.13)
Inappropriate reaction against errors	198(62.0)	73(22.9)	48(15.0)	2.70 ± 1.07
Their discrimination between wrongdoer	199(62.3)	78(24.5)	42(13.1)	2.76 ± 1.09
Their focus on wrongdoer not reason of occurring	138(74.6)	50(15.7)	31(9.7)	3.00 ± 1.01
Lack of good education for reporting errors	195(60.9)	72(22.5)	53(16.6)	2.63 ± 1.10
Lack of professional support by medical service	192(60.4)	83(26.1)	43(13.5)	2.67 ± 1.08
Lack of program for prevention	194(60.6)	73(22.8)	53(16.6)	2.65 ± 1.12
Lack of systematic control on errors	192(60.4)	81(25.5)	45(14.1)	2.63 ± 1.07
**Errors scope**				**2.68 ± 0.79**
Type of errors	191(60.2)	89(28.1)	37(11.7)	2.65 ± 0.99
Severity and significance	202(63.1)	88(27.5)	30(9.4)	2.73 ± 0.97
Lack of clear definition for medical team	188(58.8)	90(28.1)	42(13.1)	2.63 ± 0.98
**Patient’s scope**				**2.41 ± 0.87**
Their lack of notification about errors happening	198(62.2)	71(22.3)	49(15.4)	2.67 ± 1.08
Physical Critical position of patient	176(55.0)	70(21.9)	74(23.1)	2.43 ± 1.11
Death prospect	161(50.5)	75(23.5)	83(26)	2.38 ± 1.15
Low level of economic, social	152(47.5)	77(24.1)	91(28.4)	2.26 ± 1.17
Lack of following by patient’s family	156(48.9)	73(22.9)	90(28.2)	2.31 ± 1.18

In this study the relationship between other variables such as age, gender, years of service and job position were not statistically significant regarding the reasons of not reporting professional errors (Table 3).

**Table 3:** Correlations between characteristics of participants and not reporting error total score and scopes

Variable	Total score of not reporting	Medical team scope	Managers scope	Error scope	Patient scope
0.02	0.02	0.02	0.03	0.01	age
0.01	0.06	0.02	0.09	0.03	Gender
0.01	0.09	0.03	0.04	0.03	years of service
0.01	0.03	0.02	0.04	0.01	Job Position

## 4. Discussion

Patient’s safety is one of the most important dimensions of healthcare service (Marin, 2004). Medical errors can jeopardize this safety and cause mortality. Healthcare administrators consider medical errors as one of the principle problems of patient treatment (Valizadeh, Ghasemi, Najafi, Delfan & Mohsenzadeh, 2008). Although patient’s safety will be increased with reporting and recording medical errors, if the time between the error occurrence and error reporting is reduced, medical teams can achieve their goal better (Cheraghi, Nikbakhat, Nasabadi, Mohammad Nejad, Salari, & Ehsani KouhiKheyli, 2012). This study aimed to finding factors on not reporting professional errors. Therefore, factors related to errors and managers contained more important reasons than others. Different studies showed same or different results in not reporting and recording errors. In agreement to the present study, Tal, Pourreza, Sharifirad, Mohebbiand Ghazi (2010) showed that nursing groups mentioned managerial factors and fear of legal prosecution in reporting pharmacological errors. Mardani and Shahraky (2009) mentioned that managerial factors were the important reason for not reporting errors. Blegen, Vaughnand and Goode (2001) found that fear of outcome was the reason of not reporting.

Etchegaray and Throckmorton (2010) also introduced obstacles such as fear of the consequences of reporting, impaired occupational and professional identity, organizational factors and information gap as major obstacles for not reporting errors. Mirzaei, Khatony, Safari Faramani, and Sepahvand (2013) have also reported factors such as legal issues related to staff in reporting errors, concerns about labeling of inadequacy to nurses as the most common barriers of medication errors.

Some studies such as Chiange and Pepper’s in Taiwan (2006) showed that problems of reporting and managers’ obstacles are hinders of reporting errors. On the whole, all care service members share this idea that managerial factors are the most important reason for not reporting errors. However, other variables such as type of errors, severity, importance of errors, and a lack of clarity in definitions of errors are the most important factors for not reporting the errors.

Managers’ concentration on wrongdoers instead of systematic focus on the cause of errors, discrimination between wrongdoers, and inappropriate reactions were the most important factors in this study. The most prevalent reason for not reporting errors announced by Mohamadnejad et al. (2009) was the effect of professional errors on the evaluation and managers vituperation which is in agreement with this study. Though, managerial factors were the main reason for not reporting errors, Krizek (2000) also supported our results for not reporting and recording errors by medical teams such as improper reaction and legal prosecution. In several cases, fear of negative reactions, job inadequacy, patients’ negative attitudes, and legal prosecution were mentioned as the most important factors of not reporting errors which are in agreement with our results (Osborne, Blais & Hayes, 1999; Wakefield et al., 1999; Elder, Graham, Brandt, & Hickner, 2007; Anosheh, Ahmadi, Faghihzadeh, & Veys, 2007). In Mardani and Shahraky’s study (2009) such results were mentioned as the factors of not reporting and recording errors. Baghcheghi and Kouhestani (2010) found managerial factors were the cause for not reporting pharmacological errors.

However these justifications are not true about all employees, but MohammadNejad, Ehsani, Salari, Sajjadi and Hajiesmaeel Pour’s study (2013) showed that fear of yearly evaluation was the reason to hide these errors. Therefore, positive and effective communication between managers and nursing staff and also reassuring the medical staff that their medical errors and reports are confidential is necessary.

In a general classification, Hannawa, Beckman, Mazor, Paul, and Ramsey (2013) also believe that clinical, legal, ethical and communicative aspects are among the important aspects which cause tension in individuals when reporting errors.

If we completely study these results, we can understand that all factors are the same despite their different names and subcategories and with which all employees agree. Therefore, using effective and suitable strategies for deleting all factors is very important.

Kabirzadeh, Bozorgi, Moatamad, MohsaniSaravi and GholipourBaradari (2011) in their study titled “a survey of manager’s attitude about voluntary announcing professional errors” declared that most of the individuals agreed that reporting errors can increase patient’s safety, on the other hand, manager’s focus on wrongdoers without estimating other related factors ranked first in this study. It is in agreement with Tal et al.’s (2010) study. It is obvious that managers’ effective relationship with nurses is important for reporting errors without any worries to respect professional ethics. Also managers’ attempt for investigating obstacles of their own field and other fields, using systematic procedures to find results on professional errors, omitting those factors and designing systematic ways of reporting are of great importance. It should be clear why and how errors occur; we should also find weak points and try to remove those (Blegen et al., 2004).

Eadie (2012) pointed out another useful aspect of error reporting and believed that reporting professional errors may lead to better learning among medical employees and thus increase patients’ safety. For example, in the voluntary reporting system in Scotland no information about the wrongdoer is released to authorities in order to overcome personal fears about disclosure of names. In Canada, the error reporting policy has created an environment in which no information is revealed about the reporter of the error, so fear of disclosing the name of the wrongdoer or reporter of the error is eliminated as a barrier to reporting the error (J. Kalra, N. Kalra & Baniak, 2013).

Another factor that we did not mention in this study was the time consuming process of recording the errors. In this regard, the study of Lederman, Dreyfus, Matchan, Knott and Milton (2010) showed that the use of information technology-based error reporting systems is capable of reducing error reporting barriers such as the time spent for reporting the error and lack of access to appropriate sources for reporting the error.

As our results showed ‘the aspect of error’ has been mentioned by individuals as the most important factor for not reporting errors. Factors such as a lack of clear definition of errors, types of errors, importance and severity of errors were mentioned as the most important factors for not reporting errors. Shiao, Mclaws, Huang and Guo (2002) reported a lack of information about the process of reporting, misconceptions about low risks of infections and hepatitis injection as the main reasons for not reporting injuries resulted from needle hurt and other sharp objects. Education is the main source of information and clinical performance. Standard methods and acceptable ways of reporting, in all care centers and announcing other staff and students can be useful.

It is expected that the quality of healthcare will be increased as a result of correcting errors. A lack of clear definition and information about the importance of medical errors can lead to no error reporting. Wolf, Hicks and Serembus (2006) found that a lack of pharmacological information is the most important factors for not reporting pharmacological errors. Therefore, holding educational classes and retraining courses with correct educational methods, encouraging individuals to report errors and documentation of treatments can influence information about type, importance and severity of errors. Proper performance of a staff requires skills in techniques, paying attention and delivering service to patients.

Study of Bradley, Fischer and Walsh (2013) showed that 50% of students in residency-training program have not received any feedbacks about errors made by their classmates. These results support the importance of error training from the beginning of clinical education. It seems that reporting errors will also provide an appropriate background for training students on the one hand, and reduce the repeating of the error by alerting students about the errors made by others on the other hand.

In similar studies, fear of legal prosecution has been reported as the most challenging consequence of error reporting for medical teams and in this regard our results are in agreement with studies of Tal et al. (2010) and krizek (2000).

Managers must be familiar with legal principles and types of errors for removing error factors. We have to put emphasis on the length of time between the occurrence of an error and reporting it, and individuals must precisely report the error in order to prevent further problems.

Patients’ petition about the unintended violations in healthcare fields is of great importance. On the other hand, fear of punishment may be a defensive behavior that prevents reporting medical errors (Hashemi & Shool, 2009; Sozani, Bagheri, & Pourhaydari, 2007).

Patient’s complaints about probable infractions in the process of treatment are very important (Harding & Petrick, 2008; Elder, 2007). In Mardani and Shahraky’s study (2009), nurses agreed with error reporting.

In this regard, Hashemi et al.’s study (2012) also showed that nurses believe that errors should not occur due to the nature of their profession and in case of the occurrence of an error, it is necessary to report it and they should be punished for that. But Anderson, Stumpf and Schulkin’s study (2009) showed that most doctors do not tend to report their errors.

Liu et al. (2009) in their study in China categorized all reporting errors in 15 groups which were prevented. They showed that reporting errors provides more information about patient’s safety and system of reporting.

The results of this article can inform managers and medical teams about factors associated with not reporting professional errors and give them new ways for increasing knowledge about the cause and backwash effects of errors, recognizing the identity of errors, influencing the culture of reporting errors, remarking scientific and practical solutions for preventing the occurrence of errors, and extra-curricular activities in this field.

Tevlin, Doherty and Traynor (2013), in their review study, argued that for improving patient’s safety and achieving a high level of performance we should pass the present culture (Tarnishing the name and reputation, fear of blame and shame when an error occurs).

Patterson, Pace and Fincham (2013) also maintained that mere feedback about the error is not enough for creating error reporting behavior. If working condition in hospitals is the way that individuals can freely express problems related to the patient’s safety, this can contribute to the error reporting. Kagan and Barnoy (2013) pointed out that the role of senior healthcare administrators and managers can create a vision and strategy for promoting the quality improving the patient’s safety which has a key influence on the development of a safety culture and boost employees’ motivation to implement error reporting programs at the departmental and individual level.

Some limitations of this article were: negative items in the designed questionnaire according to Likert’s scale and limited number of participants (staff affiliated to Jahrom University of Medical Sciences). Therefore we have to be cautious about generalizing our results; we also suggest similar studies to be done in other hospitals with a greater sample size.

## 5. Conclusion

Educating the medical team about types of errors and how to treat them correctly is essential. As each error occurs due to a certain cause, recognizing factors which cause errors and removing them can be effective in the prevention of error re-occurrence which necessitates managers’ proper actions toward the error and the wrong doer; that is, a manager is expected to view the situation as a problem solving one, helps the wrong doer and this way provide the situation for further error reporting and preventing the re-occurrence of the error which finally results in patients’ safety.
